# The mRNA-edited form of GABRA3 suppresses GABRA3-mediated Akt activation and breast cancer metastasis

**DOI:** 10.1038/ncomms10715

**Published:** 2016-02-12

**Authors:** Kiranmai Gumireddy, Anping Li, Andrew V. Kossenkov, Masayuki Sakurai, Jinchun Yan, Yan Li, Hua Xu, Jian Wang, Paul J. Zhang, Lin Zhang, Louise C. Showe, Kazuko Nishikura, Qihong Huang

**Affiliations:** 1 Department of Tumor Microenvironment and Metastasis, The Wistar Institute, 3601 Spruce Street, Philadelphia, Pennsylvania 19104, USA; 2 University of Washington Medical Center, 1959 N.E. Pacific Street, Seattle, Washington 98195, USA; 3 Department of Radiation Oncology, Cancer Hospital of Fudan University, 270 Dong An Road, Shanghai 200032, China; 4 Institute of Cancer Stem Cell, Department of Anatomy, College of Basic Medical Sciences, Dalian Medical University, No. 9 West Section Lvshun South Road, Dalian 116044, China; 5 Department of Urology, Tongji Hospital, Tongji Medical College, Huazhong University of Sciences and Technology, Wuhan 430030, China; 6 Department of Biliary-Pancreatic Surgery, Renji Hospital, Shanghai Jiao Tong University School of Medicine, Shanghai 200127, China; 7 Department of Pathology and Laboratory Medicine, Hospital of The University of Pennsylvania, Perelman School of Medicine, Philadelphia, Pennsylvania 19104, USA; 8 Department of Obstetrics and Gynecology, Perelman School of Medicine, University of Pennsylvania, Philadelphia, Pennsylvania 19104, USA

## Abstract

Metastasis is a critical event affecting breast cancer patient survival. To identify molecules contributing to the metastatic process, we analysed The Cancer Genome Atlas (TCGA) breast cancer data and identified 41 genes whose expression is inversely correlated with survival. Here we show that GABA_A_ receptor alpha3 (Gabra3), normally exclusively expressed in adult brain, is also expressed in breast cancer, with high expression of Gabra3 being inversely correlated with breast cancer survival. We demonstrate that Gabra3 activates the AKT pathway to promote breast cancer cell migration, invasion and metastasis. Importantly, we find an A-to-I RNA-edited form of Gabra3 only in non-invasive breast cancers and show that edited Gabra3 suppresses breast cancer cell invasion and metastasis. A-to-I-edited Gabra3 has reduced cell surface expression and suppresses the activation of AKT required for cell migration and invasion. Our study demonstrates a significant role for mRNA-edited Gabra3 in breast cancer metastasis.

## Abstract (web summary)

GABRA3, a subunit of the GABA receptor, is often highly expressed in brain metastasis and breast cancers. Here, the authors demonstrated that GABRA3 activates AKT to promote breast cancer cell invasion and that the A-to-I edited form of GABRA3, specifically expressed in noninvasive breast cancers, can suppress the function of wild type GABRA3.

## Graphical abstract

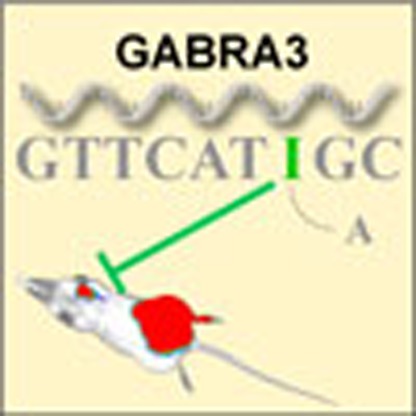

While metastasis remains the major cause of death from cancer, the critical molecular controls underlying tumour metastasis are only poorly understood. Identification of novel key regulators of metastasis and designing new ways to target and inhibit those regulators could have profound benefits to the survival of cancer-affected individuals.

Chloride functions in an electrochemical equilibrium and serves as an important signalling molecule in most cells^1^. Chloride channels are responsible for the active transport of chloride across the plasma membrane^1^. While the dysfunction of chloride transport is associated with a number of human diseases including cystic fibrosis, transport dysfunction in cancer development has not been studied extensively. Gamma-aminobutyric acid (GABA) is an inhibitory neurotransmitter^2^. GABA exerts its function through two types of GABA receptors: ionotropic receptors including the GABA_A_ and GABA_C_ receptors; and the metabotropic GABA_B_ receptor^3^. The GABA_A_ receptor is a pentamer comprised of various subunits and functions as a chloride channel^3^. The expression of the GABA_A_ receptor, the GABA transporter and the GABA transaminase has been reported to be upregulated in brain metastases of breast cancer^4^. These metastatic cells display a GABAergic phenotype similar to that of neuronal cells suggesting they use GABA for their proliferation^4^. However, whether this is the case and how the GABA_A_ receptor and its signalling pathways function in cancer development and metastasis are largely unknown.

The enzyme adenosine deaminase acting on RNA (ADAR) was originally detected as a dsRNA unwinding activity in *Xenopus* eggs and embryos^5^,^6^ and was later found to be a dsRNA-specific adenosine deaminase^7^,^8^. These discoveries opened up the previously unrecognized field of A-to-I (adenosine-to-inosine) RNA editing^9^,^10^,^11^,^12^,^13^,^14^,^15^,^16^,^17^. ADARs specifically target dsRNAs and deaminate adenosine residues to inosine via a hydrolytic deamination reaction (A-to-I RNA editing). The edited inosine residue in RNA is detected as an A-to-G change in the cDNA sequence, and the translation machinery reads inosine as guanosine, leading to alterations of codons. Interestingly, the coding region of chloride channel Gabra3, one of the subunits of GABA_A_ receptor, undergoes A-to-I editing, which results in one amino-acid change in GABA_A_ receptor alpha3 protein^18^. However, the functions of A-to-I-edited GABA_A_ receptor alpha3 in cancer development have not been studied.

Using bioinformatic analysis of breast cancer genomics data, we discovered that high expression of Gabra3 is significantly inversely correlated with breast cancer survival. We now show that overexpression of Gabra3 promotes breast cancer cell migration, invasion and metastasis. Conversely, the knockdown of Gabra3 expression suppresses cell invasion and metastasis with no detectable effect on cell proliferation. Importantly, we also show that Gabra3 is highly expressed in breast cancer tissues and cell lines but not in normal breast epithelial cells or normal breast tissue. Mechanistically, we show that (1) Gabra3 activates the AKT pathway to promote cell migration and invasion; (2) that A-to-I editing of Gabra3 occurs only in non-invasive breast cancer cells; and (3) that RNA-edited Gabra3 transdominantly suppresses the functions of unedited Gabra3 in promoting cell invasion and metastasis.

## Results

### Identification of Gabra3 in breast cancer progression

**Figure 1:**
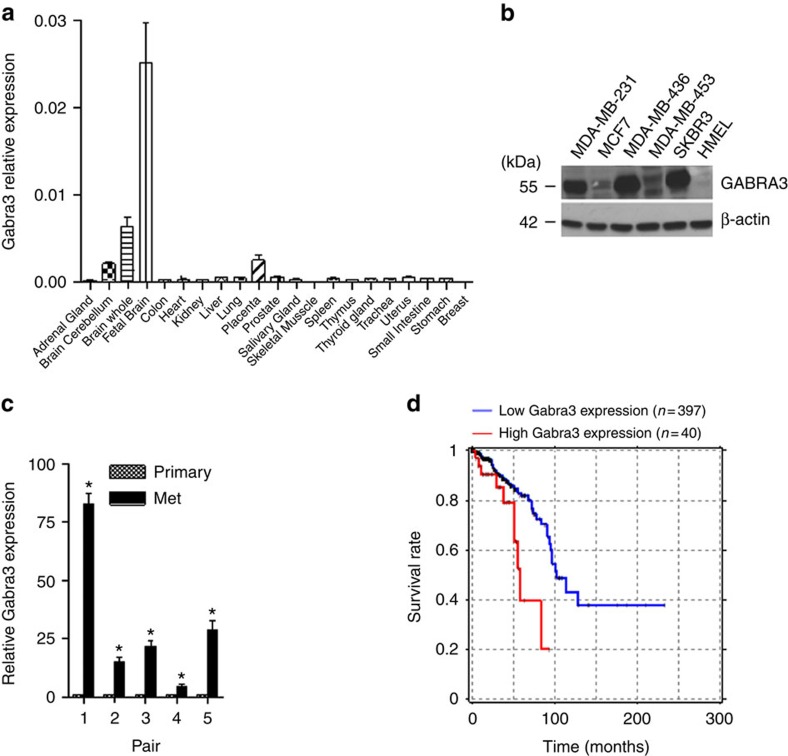
Gabra3 is highly expressed in human breast cancer cells and tissues but not in normal human breast tissues and cells. High Gabra3 expression is inversely correlated with survival in breast cancer. (**a**) Expression of Gabra3 transcript in normal human tissues. Error bars represent mean±s.d.; *n*=3. (**b**) Gabra3 protein expression in human breast cancer cell lines SKBR3, MDA-MB-453, MDA-MB-436, MDA-MB-231 and normal human breast epithelial cell HMEL. (**c**) Expression of Gabra3 transcript in paired metastatic and primary human breast cancer tissues. *P* value was determined using Student's *t*-test (*P*<0.01). Error bars represent mean±s.d.; *n*=3. (**d**) Association of high Gabra3 expression with poor survival in breast cancer (Cox regression *P*=0.001, hazard ratio=2.85).

To identify genes that are critical for breast cancer progression, we analysed The Cancer Genome Atlas (TCGA) RNA-seq data for breast cancers and normal breast tissues, as well as the associated survival data (see Methods). We identified 41 genes that met the four conditions for selection as described in Methods (Supplementary Table 1). The upregulation of 40 of them and downregulation of one (SFTBP) was associated with poor survival (Supplementary Table 1). Among the overexpressed poor prognosis genes, many had been previously shown to be significantly upregulated in cancer. For example, upregulation of telomerase expression has been shown to be critical in the development of multiple cancer types^19^. Furthermore, several transcription factors including ONECUT2, POU4F1 and NOTUM, have previously been shown to promote tumorigenesis^20^,^21^,^22^. In this study, we chose to focus on the chloride channel protein Gabra3 for the following three main reasons: it is highly expressed in cancer tissues but not in normal breast tissues; it is a cell surface molecule and thus a potential drug-targetable protein; therapeutics targeting Gabra3 are already being used in the clinics for other purposes^23^. We first determined the expression pattern of Gabra3 in a panel of normal human tissues and found that it was expressed much more strongly in adult brain tissues than in other adult organs (Fig. 1a) with the highest Gabra3 expression being in the foetal brain (Fig. 1a). Gabra3 expression is also pronounced in the cortex and thalamus at embryonic and early postnatal stages in rat^24^,^25^, indicating its role in the development of cortical plate. Immunoblotting of Gabra3 in human breast cancer cell lines indicated that it was expressed at various levels in breast cancer cell lines but not expressed in the normal human epithelial cell line HMEL (Fig. 1b). In paired human breast cancer samples, Gabra3 expression was higher in the metastatic samples than in the matched primary breast cancer samples (Fig. 1c) supporting our TCGA data survival analysis indicating that higher Gabra3 expression correlated with poorer survival (Fig. 1d). Multivariate Cox regression tests further demonstrated that Gabra3 expression associated with survival is independent of other tumour characteristics including tumour stage (Supplementary Table 2). Gabra3 expression and stage were found to be significant but independent predictors of survival in a multivariate model (Supplementary Table 2).

### Gabra3 promotes breast cancer invasion and metastasis

**Figure 2:**
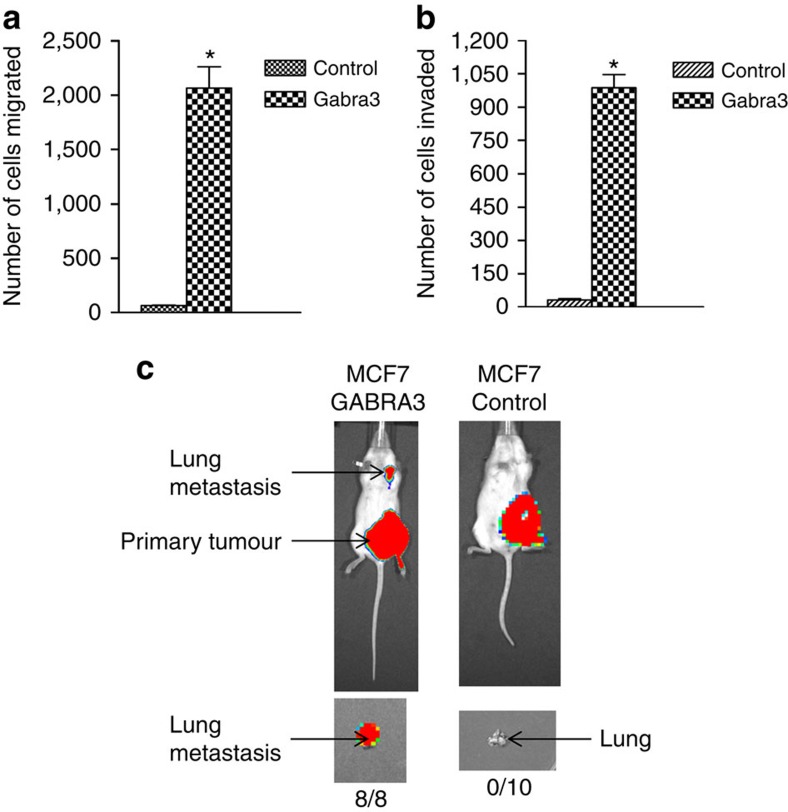
Gabra3 promotes tumour cell migration, invasion and metastasis in breast cancer. (**a**,**b**) Human breast cancer MCF7 cells stably expressing Gabra3 were subjected to migration (**a**) and invasion (**b**) assays. The numbers of migrated and invaded cells were quantified. *P* value was determined using Student's *t*-test (*P<0.001). Error bars represent mean±s.d.; *n*=3. (**c**) Luciferase-tagged human breast cancer MCF7 cells stably expressing Gabra3 or a vector control were transplanted in mouse mammary fat pads. Primary tumour and metastasis were imaged using Xenogen bioluminescence system. *P* value was determined using Fisher's exact test (*P*<0.0001).

**Figure 3:**
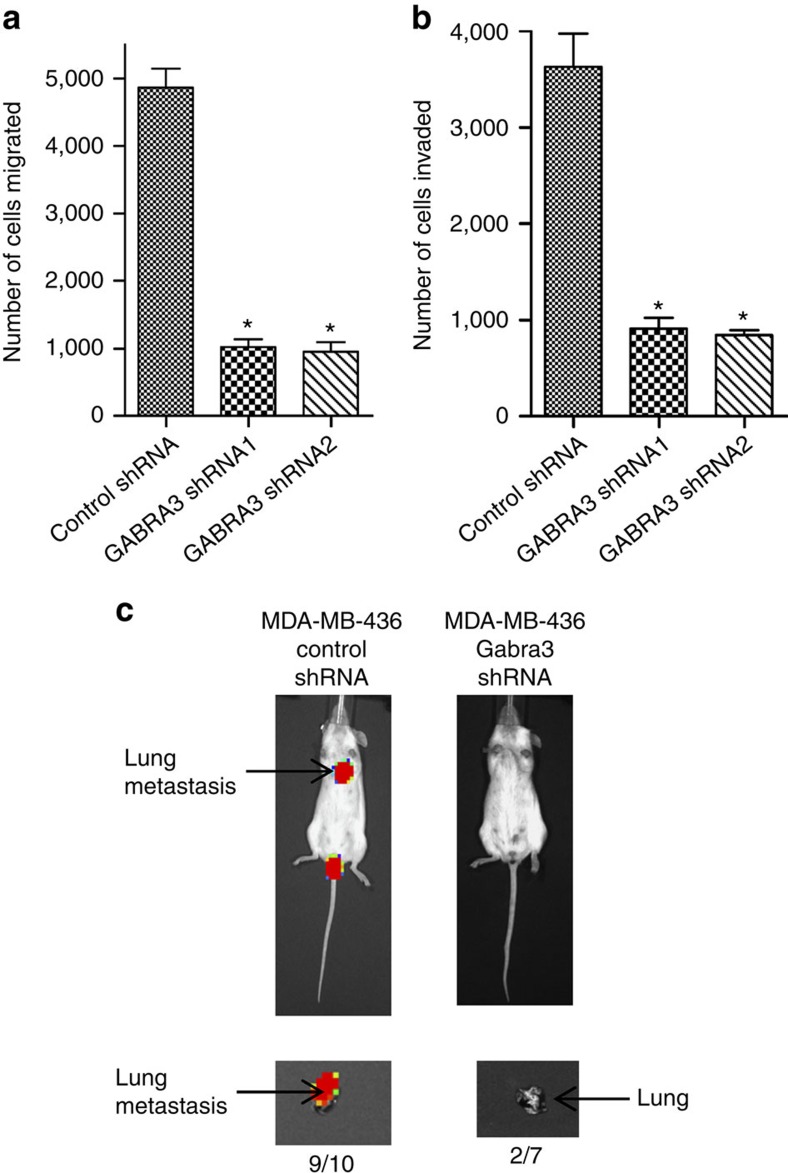
Suppression of Gabra3 expression inhibits tumour cell invasion and metastasis in breast cancer. (**a**,**b**) Human breast cancer MDA-MB-436 cells stably expressing Gabra3 shRNAs were subjected to migration (**a**) and invasion (**b**) assays. The numbers of migrated and invaded cells were quantified. *P* value was determined using Student's *t*-test (*P<0.01). Error bars represent mean±s.d.; *n*=3. (**c**) Luciferase-tagged human breast cancer MDA-MB-436 cells stably expressing Gabra3 shRNA or control shRNA were transplanted in mice. Metastasis was imaged using Xenogen bioluminescence system. *P* value was determined using Fisher's exact test (*P*=0.035).

As Gabra3 appeared to be more highly expressed in metastatic tissues than in primary tumours, we assessed the contribution of Gabra3 to cell migration and invasion. We introduced Gabra3 into human breast cancer MCF7 cells, which express endogenous Gabra3 but at low levels (Fig. 1b), and subjected these cells to migration and invasion assays. The expression of Gabra3 was confirmed by real-time PCR (Supplementary Fig. 1). MCF7 cells expressing increased Gabra3 exhibited significantly increased migration (Fig. 2a) and invasion (Fig. 2b) capabilities when compared with cells expressing a control vector. We then measured the metastasis-promoting activity of Gabra3 *in vivo*. Luciferase-tagged MCF-7 cells expressing Gabra3 or a control vector were transplanted into mice mammary fat pads. Lung metastases developed in all the mice injected with cells expressing Gabra3, whereas no metastases were observed in any of the mice injected with cells expressing the control vector (Fig. 2c), suggesting that Gabra3 expression is sufficient for metastases promotion. To determine whether Gabra3 is required for breast cancer metastasis, we introduced short hairpin RNA constructs into human breast cancer MDA-MB-436 cells expressing high levels of endogenous Gabra3 (Fig. 1b). Knockdown of Gabra3 was confirmed by real-time PCR (Supplementary Fig. 2). While the knockdown of Gabra3 in MDA-MB-436 cells did not affect cell proliferation (Supplementary Fig. 3), the migration (Fig. 3a) and invasion (Fig. 3b) capabilities of MDA-MB-436 cells were significantly reduced. Luciferase-tagged MDA-MB-436 cells expressing Gabra3 shRNAs or a control shRNA were also transplanted into mice. Nine out of ten mice injected with cells expressing a control shRNA developed lung metastasis, whereas only 2 out of 7 mice injected with cells expressing a Gabra3 shRNA developed metastasis (Fig. 3c). Taken together, these results suggested that Gabra3 is both sufficient and required for breast cancer metastasis.

### RNA-edited Gabra3 suppresses breast cancer metastasis

**Figure 4:**
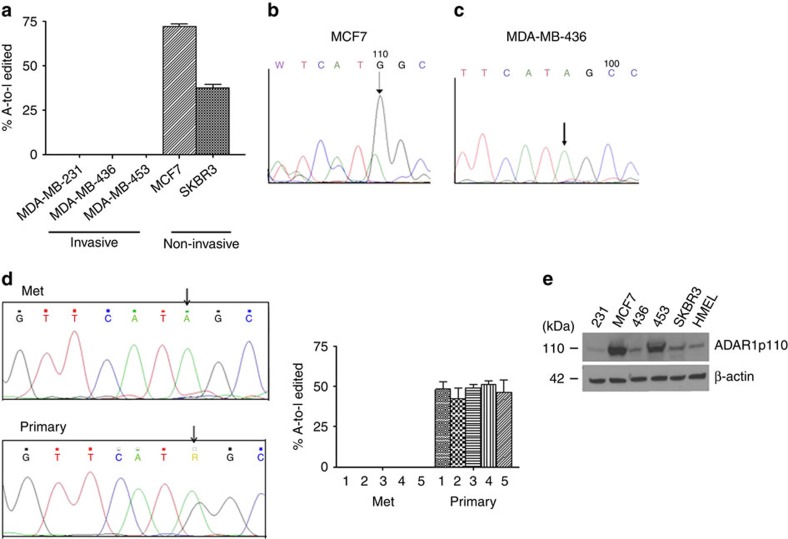
A-to-I RNA-edited Gabra3 is expressed in non-invasive human breast cancer cells. (**a**) Percentage of A-to-I-edited Gabra3 expressed in human breast cancer cell lines were determined by sequencing. (**b**) Sequencing of Gabra3 expressed in non-invasive human breast cancer MCF7. Arrow indicates the A-to-I-edited nucleotide of Gabra3. (**c**) Sequencing of Gabra3 expressed in invasive human breast cancer MDA-MB-436 cells. Arrow indicates the nucleotide of adenosine of the unedited Gabra3. (**d**) Percentage of A-to-I-edited Gabra3 expressed in paired human breast cancer primary and metastatic samples was determined by sequencing. (**e**) Expression of RNA-editing enzyme ADAR1p110 in human breast cancer cells and normal human breast epithelial cells. All error bars in the figure represent the mean±s.d. of three independent experiments.

Gabra3 has been shown to be A-to-I RNA edited at the so-called I/M site in the human brain, resulting in the substitution of an isoleucine with methionine^18^,^26^. We asked whether it is similarly edited in human breast cancer tissue by directly sequencing Gabra3 mRNA in human breast cancer cell lines. We found RNA editing of Gabra3 only in the non-invasive MCF7 and SKBR3 breast cancer cells (Fig. 4a,b) but not in the invasive cell lines MBA-MD-231, MBA-MD-436 and MDA-MB-453 (Fig. 4a,c). Comparing paired primary and metastatic human breast cancer samples, we found that the metastatic samples did not express RNA-edited Gabra3, whereas primary tumour samples did (Fig. 4d). Two enzymes are responsible for A-to-I editing, ADAR1 and ADAR2 (ref. 10). Furthermore, there are two isoforms of ADAR1 generated by usage of different promoters and alternative splicing; ADAR1p150 and ADAR1p110 (ref. 10). We determined ADAR1 and ADAR2 expression in these cell lines. While ADAR1p110 was expressed in all the breast cancer cell lines we tested along with the normal human epithelial cell HMEL (Fig. 4e), neither ADAR1p150 nor ADAR2 was detected in any of the cell lines. These results suggest that ADAR1p110 is the enzyme responsible for editing of Gabra3 mRNAs.

**Figure 5:**
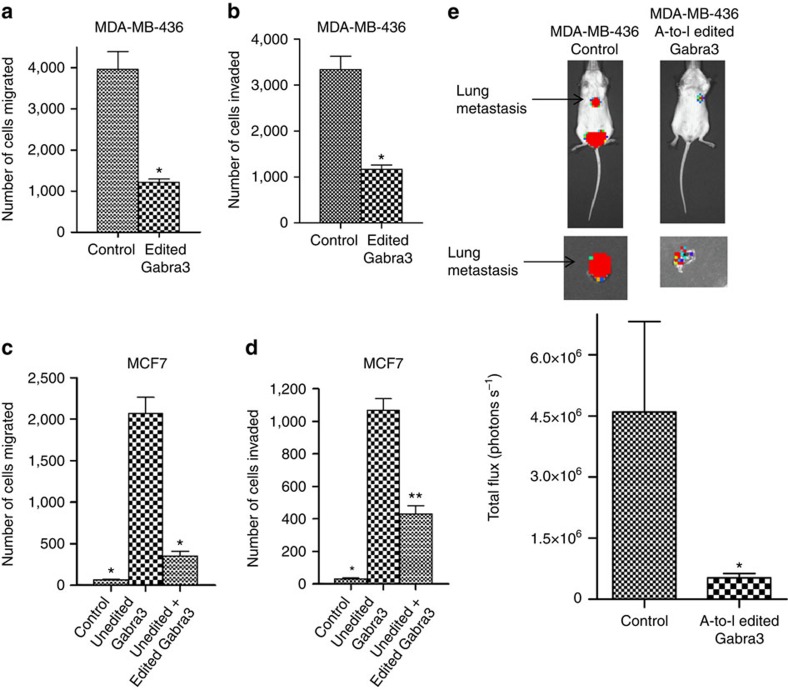
A-to-I RNA-edited Gabra3 suppresses tumour cell migration, invasion and metastasis in breast cancer. (**a**,**b**) Human breast cancer MDA-MB-436 cells stably expressing RNA-edited Gabra3 were subjected to migration (**a**) and invasion (**b**) assays. The numbers of migrated and invaded cells were quantified. *P* value was determined using Student's *t*-test (*P<0.001). (**c**,**d**) Human breast cancer MCF7 cells stably expressing a control vector, or unedited Gabra3, or unedited Gabra3 and RNA-edited Gabra3, were subjected to migration (**c**) and invasion (**d**) assays. The numbers of migrated and invaded cells were quantified. *P* value was determined using Student's *t*-test (*P<0.001, **P<0.01). (**e**) Luciferase-tagged human breast cancer MDA-MB-436 cells stably expressing RNA-edited Gabra3 or a vector control were transplanted in mice. Luciferase signals of metastasis were quantified using Xenogen bioluminescence system. *P* value was determined using Student's *t*-test (*P*<0.05). All error bars in the figure represent the mean±s.d. of three independent experiments.

To further assess the functions of A-to-I-edited Gabra3 in breast cancer cells, we then introduced RNA-edited Gabra3 carrying an A-to-G mutation at the I/M site into MDA-MB-436 cells that endogenously express only unedited Gabra3, and subjected these cells to migration and invasion assays. The migration (Fig. 5a) and invasion (Fig. 5b) capabilities of the cells expressing edited Gabra3 message were significantly reduced when compared with the cells expressing a control vector. Similar phenotypes were also observed in MCF7 cells where stable expression of unedited Gabra3 promoted cell migration (Fig. 5c) and invasion (Fig. 5d) and the expression of edited Gabra3 reversed the migratory (Fig. 5c) and invasive phenotypes (Fig. 5d). To assess the suppressive effects of ADAR1 on migration and invasion, we ectopically expressed ADAR1 in invasive cell lines MBA-MD-231, MBA-MD-436 and MDA-MB-453 (Supplementary Fig. 4). Increased ADAR1 expression significantly decreased migration (Supplementary Fig. 5) and invasion (Supplementary Fig. 6) in these cell lines. To determine whether RNA-edited Gabra3 had similar effects *in vivo*, luciferase-tagged MDA-MB-436 cells expressing A-to-I-edited Gabra3 or a control vector were injected into mice. The luciferase signal indicating lung metastasis was significantly reduced in mice transplanted with the cells expressing RNA-edited Gabra3 compared with mice with the cells expressing a control vector (Fig. 5e). These results further support our hypothesis that A-to-I-edited Gabra3 has an opposing function to unedited Gabra3 with its presence suppressing rather than inducing invasion and metastasis in breast cancer.

### RNA-edited Gabra3 suppresses AKT activation

**Figure 6:**
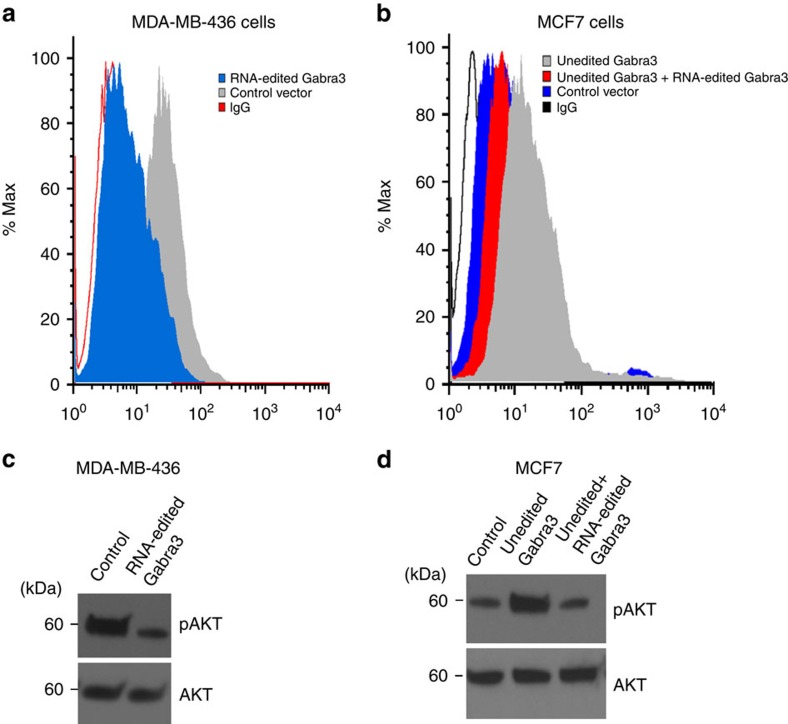
A-to-I RNA-edited Gabra3 reduces Gabra3 expression on cell surface and suppresses AKT activation. (**a**) Representative flow cytometry histogram overlay of Gabra3 surface expression in MDA-MB-436. Human breast cancer MDA-MB-436 cells expressing RNA-edited Gabra3 (blue), or a control vector (grey), were subjected to FACS analysis using a Gabra3 antibody or control IgG. (**b**) Representative flow cytometry histogram overlay of Gabra3 surface expression in MCF7 cells. Human breast cancer MCF7 cells stably expressing a control vector (blue), or unedited Gabra3 (grey), or both unedited Gabra3 and RNA-edited Gabra3 (red), were subjected to FACS analysis using a Gabra3 antibody or control IgG. The expression of RNA-edited Gabra3 reverses the phenotypes of wild-type Gabra3. (**c**) Phosphorylated and total AKT in human breast cancer MDA-MB-436 cells expressing RNA-edited Gabra3, or a control vector, were determined by immunoblotting. (**d**) Phosphorylated and total AKT in human breast cancer MCF7 cells stably expressing a control vector, or unedited Gabra3, or both unedited Gabra3 and RNA-edited Gabra3, were determined by immunoblotting.

It has previously been shown that A-to-I-edited Gabra3 affects the intracellular trafficking of Gabra3 (ref. 26), that is critical for the localization of Gabra3 on cell membrane. We determined the cell surface expression of Gabra3 using fluorescence-activated cell sorting (FACS) analysis in MDA-MB-436 cells expressing RNA-edited Gabra3 or a control vector and MCF7 cells expressing unedited Gabra3, both edited and unedited Gabra3, or a control vector. We found that the A-to-I-edited Gabra3 had reduced the expression on the cell surface in MDA-MB-436 cells (Fig. 6a). Overexpression of unedited Gabra3 in MCF7 cells increased the expression of Gabra3 on cell surface (Fig. 6b), while expression of RNA-edited Gabra3 reversed this phenotype (Fig. 6b).

The downstream signalling pathways that mediate the functions of Gabra3 in tumour invasion and metastasis are unknown, but since the AKT pathway is critical in both breast cancer metastasis and therapy resistance^27^,^28^,^29^, we determined the effect of Gabra3 on AKT activation. MCF7 cells stably expressing unedited Gabra3 were treated with a specific pan-AKT inhibitor MK-2206 and the cells were subjected to migration and invasion assays. MK-2206 reversed the migratory and invasive phenotypes of MCF7 cells stably expressing unedited Gabra3 in a dose-dependent manner (Supplementary Fig. 7). This suggests that Gabra3 promotes migration and invasion at least in part through the activation of AKT signalling. Ectopic expression of RNA-edited Gabra3 reduced the level of phosphorylated AKT but did not affect total AKT levels in MDA-MB-436 cells (Fig. 6c). Conversely, overexpression of unedited Gabra3 increased the levels of phosphorylated AKT in MCF7 cells (Fig. 6d) and this could be subsequently reversed by the overexpression of A-to-I-edited Gabra3 (Fig. 6d). Other pathways such as epidermal growth factor receptor (EGFR) and extracellular signal-regulated kinase (ERK) that are connected to the AKT pathway were not activated by Gabra3 (Supplementary Fig. 8). To determine how edited Gabra3 affects the functions of unedited Gabra3, we introduced unedited or edited Gabra3 independently, or both together in a 1:1 ratio into the human mammary epithelial cell line, MCF10A, which does not express endogenous Gabra3 (Supplementary Fig. 9). We determined the cell surface expression of Gabra3 using FACS analysis. We found that cell surface expression of Gabra3 in MCF10A cells expressing A-to-I-edited Gabra3 was significantly less than that in MCF10A cells expressing unedited Gabra3 (Supplementary Fig. 10). Furthermore, cell surface expression of Gabra3 in MCF10A cells expressing edited and unedited Gabra3 in a 1:1 ratio was reduced by more than 50% when compared with MCF10A cells expressing unedited Gabra3 (Supplementary Fig. 10). These results suggest that RNA-edited Gabra3 potentially has dominant negative effects in these cells. AKT activation by Gabra3 was also reduced by more than 50% (Supplementary Fig. 11). To identify in which aspect of metastasis Gabra3 plays a role, we also determined whether Gabra3 is involved in epithelial–mesenchymal transition (EMT) process. Gabra3 expression in MCF10A cells did not significantly change the expressions of epithelial and mesenchymal markers (Supplementary Fig. 12), suggesting it is not involved in the EMT process. Since stem cells play critical roles in the metastasis process^30^,^31^, we also determined whether Gabra3 affects stem cell populations. FACS analysis indicated that unedited Gabra3 expression significantly increased stem cell population (CD44 high/CD24 low) in MCF10A cells from 8.14 to 59.1%, whereas RNA-edited Gabra3 has no effect in promoting stem cell populations (Supplementary Fig. 13), indicating that Gabra3 may promote metastasis by increasing the CD44 high/CD24 low stem cell population. These results suggest that unedited Gabra3 activates AKT pathways, while RNA-edited Gabra3 both reduces cell surface Gabra3 expression and suppresses AKT activation.

## Discussion

Our analysis of the TCGA breast cancer data uncovered a number of genes that are associated with survival. Since patient survival involves two major factors: resistance to therapy and metastasis, we were careful to focus our study on genes significantly associated with survival in our bioinformatics study and that also showed an impact on metastasis when silenced and thus selected Gabra3 for further analysis based on its potential as a druggable target. The Gabra3-regulated cellular pathway we identified functions in both therapy resistance and metastasis. Among the genes we found that are associated with survival, most are currently not druggable. In contrast, Gabra3 is a cell surface receptor and already has a few regulators approved by FDA and used in the clinics for other purposes.

Gabra3 is normally expressed in neuronal tissues but not breast epithelial cells. However, there is precedence for neuronal proteins becoming aberrantly expressed in cancer and contributing to metastasis. Importantly, there is also precedence for therapeutic targeting of these aberrantly expressed neuronal proteins for cancer treatment as with the glutamate receptor GRM1, which is aberrantly overexpressed in melanoma^32^. Riluzole, an FDA-approved drug that inhibits the release of glutamate and is used for the treatment of amyotrophic lateral sclerosis has been shown to suppress GRM1 and the glutamate neurotransmission pathway, and decrease melanoma progression^33^,^34^,^35^. These results indicate that a gene that is specifically expressed in one tissue but aberrantly expressed in tumours of another tissue can play important roles in tumour development, and can thus serve as important therapeutic targets. Modulations of GABA_A_ receptors are associated with sedation, ataxia, amnesia, anxiolytic and sleep activity^36^,^37^ and there are a few GABA_A_ receptor modulators currently used in clinic. Flumazenil is a FDA-approved small molecule negative modulator of GABA_A_ receptors, which targets GABRA1, 2, 3 and 5 (ref. 38). It is used to treat idiopathic hypersomnia and improve vigilance. It is worthwhile to determine whether flumazenil and other chloride channel blockers can be used as therapeutics to also treat breast cancer metastasis.

Our study opens up a number of questions regarding the role of GABA and its receptors in breast cancer metastasis. Whether GABA is required for breast cancer cells to proliferate in metastatic sites, particular in brain, has not been elucidated. We have shown that Gabra3 activates the AKT pathway that is critical to both metastasis and therapy resistance^27^,^28^,^29^. AKT activation promotes cell motility, invasion and metastasis by activating downstream molecules including FAK, MMPs and so on^39^,^40^. Interestingly, PI3K/AKT/mTOR pathway activation is also required for the viability and maintenance of breast cancer stem cells^41^. Our study showed that unedited Gabra3 expression activated AKT and increased stem cell populations (Supplementary Figs 11 and 13). How the AKT pathway is regulated by Gabra3 remains to be further studied. Although it has been shown that GABA_A_ receptor regulates EGFR activation in prostate cancer^42^, our results indicate that this is not the case in breast cancer cells (Supplementary Fig. 8). Although more than half of the basal-like breast cancers express high levels of EGFR^43^, AKT activation in basal-like breast cancer is less common^44^. This suggests that these two pathways may have different functions. It is possible that Gabra3 regulates signalling molecules upstream of AKT pathway, which consequently activate AKT. Further characterization of Gabra3 signalling pathways will enhance our understanding of its functions in cancer development.

To our knowledge, this is the first time to report that RNA editing plays critical roles in breast cancer progression, invasion and metastasis and also that RNA editing may be a potential therapeutic target. Interferons upregulate the expression of ADAR1, which we have shown is responsible for Gabra3 editing in breast cancer cells^45^,^46^,^47^. Recombinant interferon-β (IFN-β) has been shown in clinical trials to improve clinical benefits and overall survival in metastatic breast cancer patients with minimal residual disease after chemotherapy or with disseminate disease non-progressing during endocrine therapy^48^,^49^,^50^. Thus, the combination of targeting Gabra3 function and upregulating A-to-I editing of Gabra3 mRNAs may have the potential to further improve therapeutic effects as combination therapies.

## Methods

### TCGA data analysis

RNA-seq of TCGA breast cancer and normal breast tissue data set were compared using the EdgeR method to find genes significantly dysregulated in cancer. For the cancer group, we also performed Cox regression analysis to find genes significantly associated with breast cancer survival. Genes were identified that satisfied the following four conditions: (1) genes were significantly differentially expressed in samples from breast cancer compared with samples from normal breast tissue (false discovery rate (FDR)<5%); (2) the difference in expression was at least five-fold; (3) genes were significantly associated with survival (*P*<0.05); and (4) the direction of gene expression difference was to be aligned with the direction of survival, that is, genes with higher expression in cancer had to have their higher expression associated with poor survival, and vice versa. The following available data were used as variables in a Cox regression model: GABRA3 expression, cancer subtypes ER, PR, HER2, TNBC, T, N, M, stage. Three types of Cox regression models were fitted: using each variable independently, using each variable combined with GABRA3 expression levels and by combining all variables. The significance of association of GABRA3 expression with survival in multivariate Cox regression models indicated this association is independent from other variables.

### Cell culture

MCF7, MCF10A, MDA-MB-231, MDA-MB-436, MDA-MB-453 and SKBR3 were purchased from American Type Culture Collection (ATCC). HMEL was provided by Dr Robert Weinberg, Whitehead Institute. All cell lines were tested and free of mycoplasma contamination (MycoAlert Mycoplasma Detection Kit, Lonza). MCF7, MDA-MB-231, MDA-MB-436, MDA-MB-453 and SKBR3 cells were grown in DMEM supplemented with 10% foetal bovine serum (FBS), 100 units per ml penicillin and 100 μg ml^−1^ streptomycin. MCF10A cells were cultured in defined mammary epithelial basal media (CC-3151, MEBM Bullet Kit, Lonza, Inc.) supplemented with 50 μl of 1 mg ml^−1^ cholera toxin and 2.5% FBS. HMEL cells were cultured in mammary epithelial growth media (CC-3051, MEGM, Lonza, Inc.).

### Lentivirus transfection and transduction

To generate MCF7 and MCF10A cells stably overexpressing Gabra3, full-length human Gabra3 was amplified by PCR with F-5'-atgataatca cacaaacaag tcactg-3' and R-5'-CTACTGTTTGCGGATCATGCC-3' primers and cloned into a lentiviral vector. Lentivirus was produced by co-transfecting subconfluent human embryonic kidney (HEK) 293T cells with Gabra3 expression plasmid or vector along with packaging plasmids pMDLg/pRRE and RSV-Rev) using Lipifectamine 2,000 as previously described^51^,^52^. Infectious lentiviruses were collected 48 h after transfection, centrifuged to remove cell debris and filtered through 0.45 μm filters (Millipore). MCF7 and MCF10A cells were transduced with the Gabra3 lentivirus. Efficiency of overexpression was determined by real-time PCR. To generate stable ADAR1 expressing breast cancer cell lines, cells were infected with ADAR1 lentivirus as described above. Efficiency of overexpression was determined by real-time PCR. MDA-MB-436 expressing Gabra3 shRNA (Sigma) or control shRNA (Sigma) were established using vector-based shRNA technique. The lentiviruses were processed as described above and transduced into MDA-MB-436 cells. The knockdown efficiency was determined real-time PCR. RNA-edited Gabra3 (A–I) was generated using the QuikChange II XL Site-Directed Mutagenesis Kit (Agilent), cloned into a lentivirus vector and transduced MCF7 expressing Gabra3 or MDA-MB-436 cells. MCF10A cells were transfected with different amount of unedited Gabra3 and RNA-edited Gabra3 cDNA using Lipofectamine 2,000 and cells were collected after 48 h.

### Tumour transplantation in mice

The MCF7 or MDA-MB-436 human breast cancer cell lines stably expressing Firefly Luciferase gene with Gabra3, or Gabra3 shRNA, or control vector, or RNA-edited Gabra3 (A-to-I) were routinely maintained at 37 °C in a humidified atmosphere of 5% CO_2_ and 95% air in DMEM medium supplemented with 10% FBS. For orthotopic injections, MCF7 (7 × 10^6^ cells per mouse) were transplanted into the mammary fat pads of the female SCID mice (6–8 weeks old). A slow-release pellet of 17β-estradiol (1.7 mg, 90-day release; Innovative Research of America, Sarasota, FL) was implanted subcutaneously in the dorsal interscapular region before the transplantation of MCF7 cells. MDA-MB-436 (1 × 10^6^) were suspended in 100 μl of PBS and injected in the lateral tail vein of 6–8 weeks old NOD/SCID mice. Mice bearing luciferase positive tumours were imaged by IVIS 200 Imaging system (Xenogen Corporation, Hopkinton, MA). Bioluminescent flux (photons per second per steradian per centimeter square ) was determined for the primary tumours and lung metastasis. Animal experiment protocols were approved by the Institutional Animal Care and Use Committee (IACUC) of the Wistar Institute. Animal procedures were conducted in compliance with the IACUC.

### Transwell migration and invasion assay

*In vitro* cell migration assays were performed as described previously^53^,^54^ using trans-well chambers (8 μM pore size; Costar). Cells were allowed to grow to subconfluency (∼75–80%) and were serum-starved for 24 h. After detachment with trypsin, cells were washed with PBS, resuspended in serum-free medium and 250 μl cell suspensions (2 × 10^5^ cells per ml) was added to the upper chamber. Complete medium was added to the bottom wells of the chambers. The cells that had not migrated were removed from the upper face of the filters using cotton swabs, and the cells that had migrated to the lower face of the filters were fixed with 5% glutaraldehyde solution and stained with 0.5% solution of Toluidine Blue in 2% sodium carbonate. Images of three random × 10 fields were captured from each membrane and the number of migratory cells was counted. The mean of triplicate assays for each experimental condition was used. Similar inserts coated with Matrigel were used to determine invasive potential in the invasion assay. To assess the effect of AKT kinase inhibitor MK2206 on MCF7-Gabra3 cell migration and invasion, assays were performed as described above in the presence of different concentrations of the inhibitor or control dimethylsulphoxide.

### RNA isolation and real-time PCR analysis

Total RNA was extracted from cell lines, using Trizol total RNA isolation reagent (Invitrogen), according to the manufacturer's specifications and treated with Turbo DNase (Ambion). cDNA was synthesized from total RNA (0.5 μg) using random hexamers with TaqMan cDNA Reverse Transcription Kit (Applied Biosystems). Gene primers (Gabra3 F-5′-GACCACGCCCAACAAGCT-3′; R-5′-AGCATGAATTGTTAACCTCATTGTATAGA-3′; GAPDH-F-5′-GAAGGTGAAGGT CGGAGTCAAC; R-5′-CAGAGTTAAAAGCAGCCCTGGT-3′) were designed using Primer Express v3.0 Software and real-time PCR was performed using SYBR Select Master Mix (Applied Biosystems). All reactions were carried out on the 7500 Fast Real-Time PCR System (Applied Biosystem). The average of three independent analyses for each gene and sample was calculated using the ΔΔ threshold cycle (Ct) method and was normalized to the endogenous reference control gene GAPDH.

### Western blotting

Standard methods were used for western blotting. Cells were lysed in lysis buffer and total protein contents were determined by the Bradford method. Thirty μg of proteins were separated by SDS–polyacrylamide gel electrophoresis under reducing conditions and blotted onto a polyvinylidene difluoride membrane (Millipore). Membranes were probed with specific antibodies. Blots were washed and probed with respective secondary peroxidase-conjugated antibodies, and the bands visualized by chemoluminescence (Amersham Biosciences). All the primary antibodies were used at 1:1,000 dilution and secondary antibodies at 1:5,000 dilution. The following antibodies were used: Rabbit polyclonal Gabra3 (Santa Cruz Biotechnology), mouse monoclonal ADAR1 (Santa Cruz Biotechnology), Rabbit polyclonal AKT, pAKT, ERK, pERK, EGFR, pEGFR, N-Cadherin, ZO1 (Cell Signaling Technology), mouse monoclonal E-cadherin, Vimentin (Cell Signaling Technology), Fibronectin (Thermo Scientific), β-Catenin (BD Bioscience), β-actin (Sigma-Aldrich) and secondary peroxidase conjugated (GE healthcare). Uncropped scans of the immunoblots were shown in Supplementary Fig. 14.

### Flow cytometry

For surface FACS (to detect surface protein expression), cells at 80% confluence were washed with PBS and collected with Versene (Sigma-Aldrich) for 15 min at 37 °C. Cells were centrifuged, resuspended in FACS buffer (0.5% BSA in PBS) and incubated with human Gabra3 (Antibodies online) or IgG isotype control (Life Technologies) antibodies at a 1:50 dilution, for 60 min at 4 °C, then washed twice in FACS buffer. For detection of Gabra3, cells were incubated with goat anti-rabbit Alexa Fluor 488 (Invitrogen, A-11008) for 30 min at 4 °C, and washed twice in FACS buffer. To evaluate cell surface expression of CD44 and CD24, 1 × 10^5^ cells were incubated with FITC-labelled mouse anti-human CD44 (Clone G44-26, BD Pharmingen) and Alexa Fluor 647-labelled mouse anti-human CD24 (Clone ML5, BD Pharmingen,), along with respective controls for 45 min at 4 °C in the dark. After incubation, cells were washed and resuspended in 500 μl of FACS buffer. Cells were analysed on a BD FACS Calibur cell analyzer (BD Biosciences). The FACS data were analysed with FlowJo software (Tree Star, Inc.). No-antibody and single-antibody controls were used to compensate the sample readings and for designating quadrants.

### RNA isolation and Gabra3 RNA editing analysis

Total RNA was extracted from cell lines and human breast cancer tissues using Trizol total RNA isolation reagent (Invitrogen), according to the manufacturer's specifications and treated with Turbo DNase (Ambion). All human samples and information were collected with written and signed informed consent and the research was approved by the institutional review board committee of the Wistar Institute. cDNA was synthesized from 1 μg of total RNA with Gabra3 gene-specific primer 5′-TTCAGTGTCCTTGGCCAGGTT-3′ using SuperScript III reverse transcriptase (Invitrogen). We aimed for high sequence quality thus performed nested PCR. PCR primers were designed within edited site to generate amplicons of the expected size only from mRNA but not from genomic DNA. First PCR product was amplified using first-strand cDNA templates and Gabra3 forward (5′-TCACAAGTGTCGTTCTGGCTCA-3′) and reverse primes (5′-TTCAGTGTC CTTGGCC AGGTT-3′). Second PCR was performed using first PCR product and Gabra3 forward (5′-CAAGTGTCGTTCTGGCTCAACA-3′) and reverse (5′-AGT GTCCTTG GCCAGGTTGAT-3′) primers. The resulting PCR fragments were purified using QIAquick gel extraction kit (Qiagen). The level of RNA editing is assessed by direct sequencing of each purified PCR product using the reverse primer used for Second PCR amplification. The percentage of A-to-I editing was determined by dividing the height of G peak at the editing site by the height of A peak plus G peak from the sequencing chromatogram.

## Additional information

**How to cite this article:** Gumireddy, K. *et al*. The mRNA-edited form of GABRA3 suppresses GABRA3-mediated Akt activation and breast cancer metastasis. *Nat. Commun.* 7:10715 doi: 10.1038/ncomms10715 (2016).

## Supplementary Material

Supplementary Information Supplementary Figures 1-14 and Supplementary Tables 1-2
