# Supplementary material for: The mRNA-edited form of GABRA3 suppresses GABRA3-mediated Akt activation and breast cancer metastasis
Source: Nat Commun. 2016 Feb 12;7:10715. doi: 10.1038/ncomms10715 (PMC4754346; doi:10.1038/ncomms10715)
Supplement: Supplementary Information — Supplementary Figures 1-14 and Supplementary Tables 1-2 [file ncomms10715-s1.pdf]

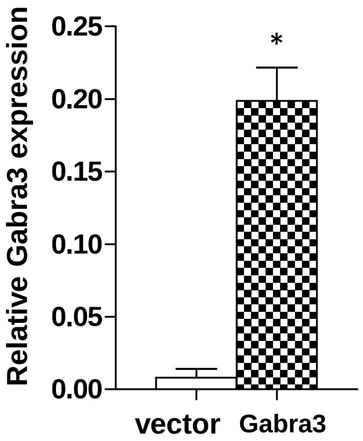

Supplementary Figure 1. Gabra3 expression is increased in MCF7 cells ectopically expressing a Gabra3 cDNA Gabra3 expression in MCF7 cells expressing Gabra3 or a control vector were determined by qRT-PCR. P value was determined using student's t test (*p<0.001). Error bars represent the mean ± s.d of three independent experiments.

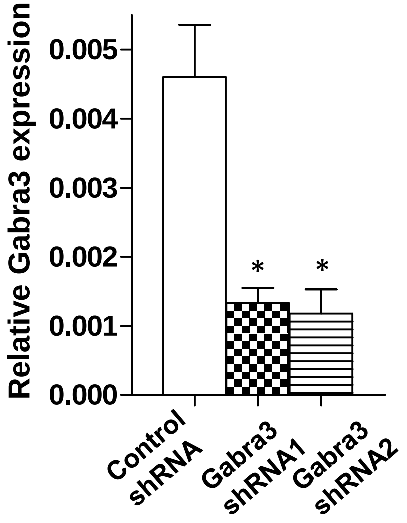

Supplementary Figure 2. Gabra3 expression is decreased in MDA-MB-436 expressing Gabra3 shRNAs
Gabra3 expression in MDA-MB-436 cells expressing Gabra3 shRNA1, or Gabra3 shRNA2, or a control shRNA were determined by qRT-PCR. P value was determined using student's t test (*p<0.001). Error bars represent the mean ± s.d of three independent experiments.

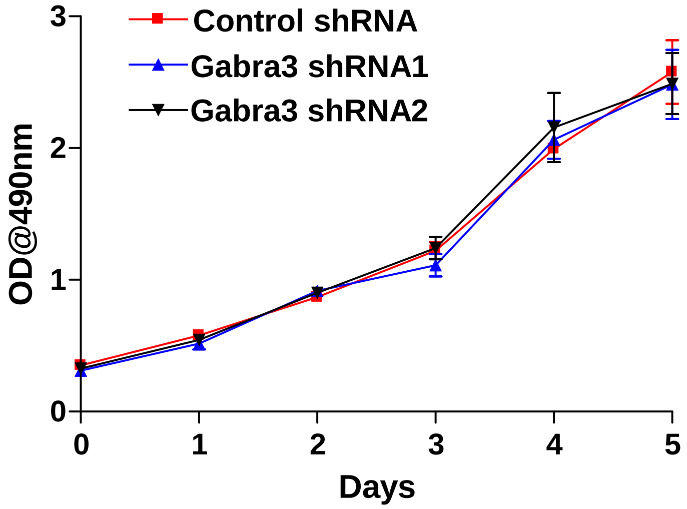

Supplementary Figure 3. Knockdown of Gabra3 did not affect cell proliferation in MDA-MB-436 cells
MDA-MB-436 cells expressing Gabra3 shRNA1, or Gabra3 shRNA2, or a control shRNA, were subjected to MTT assay. P value was determined using student's t test ($p>0.05$). Error bars represent the mean ± s.d of three independent experiments.

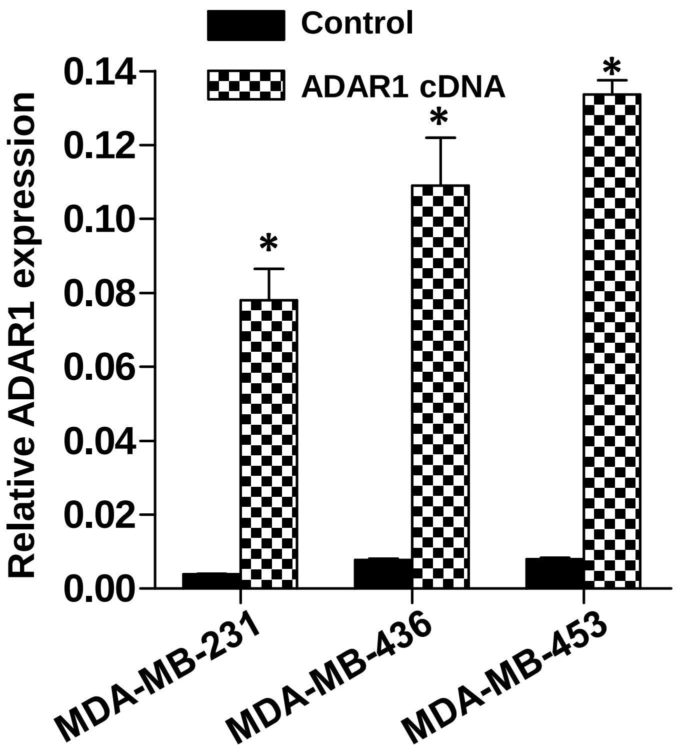

Supplementary Figure 4. ADAR1 expression is increased in human breast cancer cells ectopically expressing an ADAR1 cDNA ADAR1 expression in MBA-MD-231, MBA-MD-436, and MDA-MB-453 cells that ectopically expressed ADAR1 or a vector control. P value was determined using student's t test (*$p<0.005$). Error bars represent the mean ± s.d of three independent experiments.

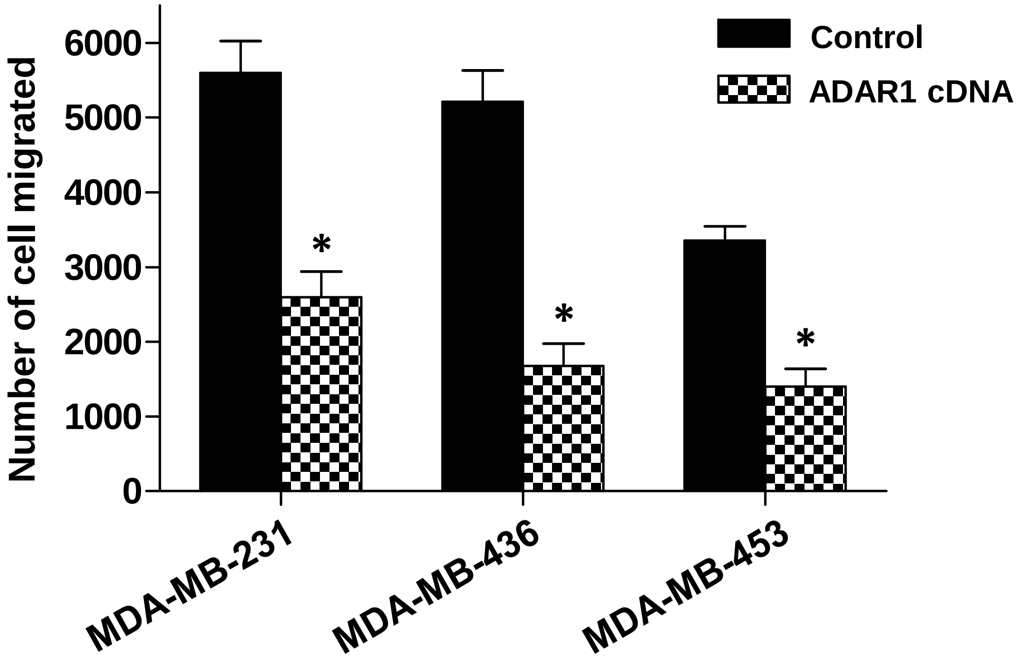

Supplementary Figure 5. Increased ADAR1 expression in these invasive cell lines significantly decreased cell migration MBA-MD-231, MBA-MD-436, and MDA-MB-453 cells that ectopically expressed ADAR1 or a vector control were subjected to migration assay. P value was determined using student's t test (*p<0.01). Error bars represent the mean ± s.d of three independent experiments.

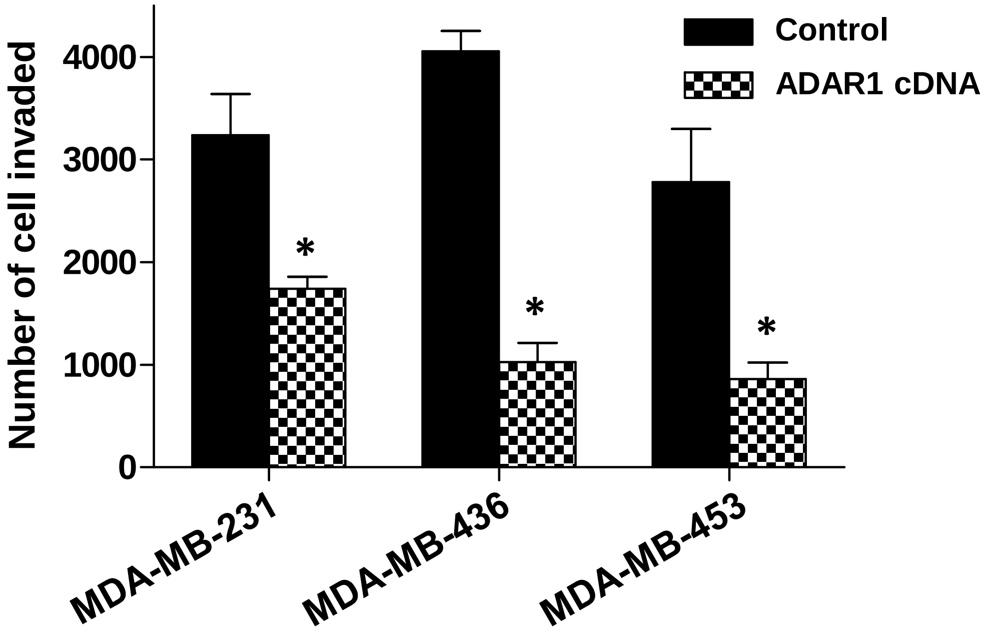

Supplementary Figure 6. Increased ADAR1 expression in these invasive cell lines significantly decreased cell invasion MBA-MD-231, MBA-MD-436, and MDA-MB-453 cells that ectopically expressed ADAR1 or a vector control were subjected to invasion assay. Increased ADAR1 expression in these invasive cell lines significantly decreased cell invasion. P value was determined using student's t test (*$p<0.05$). Error bars represent the mean ± s.d of three independent experiments.

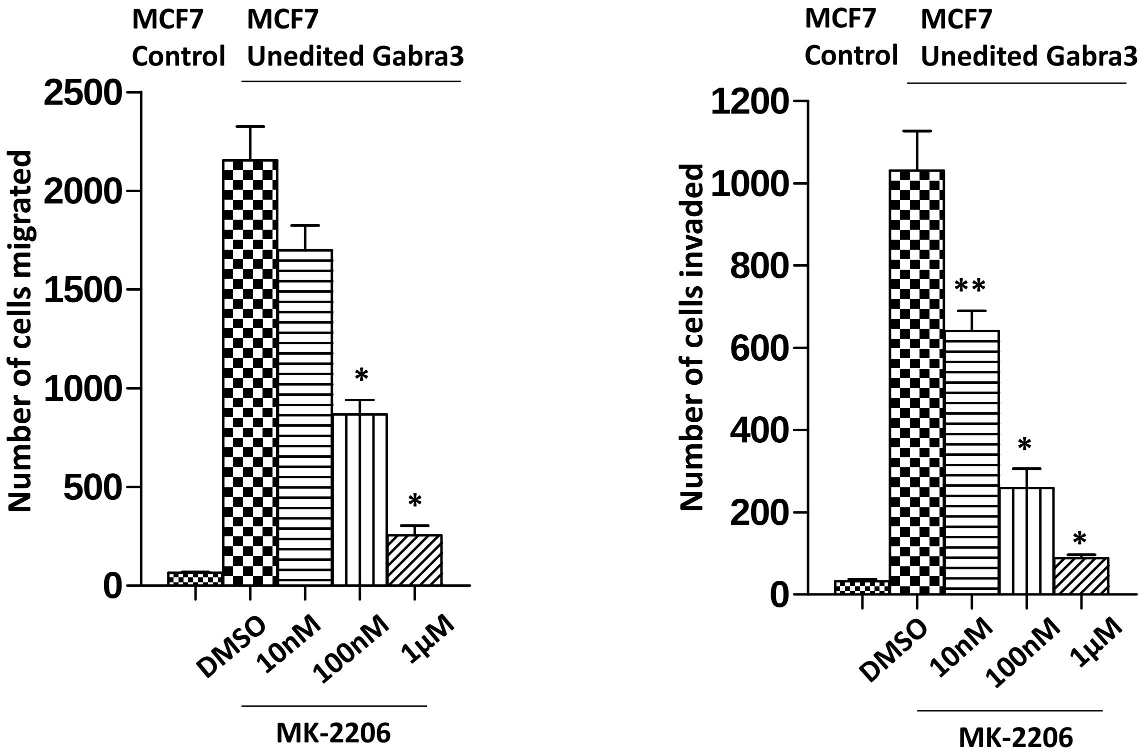

Supplementary Figure 7. AKT inhibitor MK-2206 suppressed cell migration and invasion MCF7 cells stably expressing unedited Gabra3 were treated with a pan-AKT inhibitor MK-2206 and cells were subjected to migration (A) and invasion (B) assay. P value was determined using student's t test (*p<0.005, **p<0.05). Error bars represent the mean ± s.d of three independent experiments.

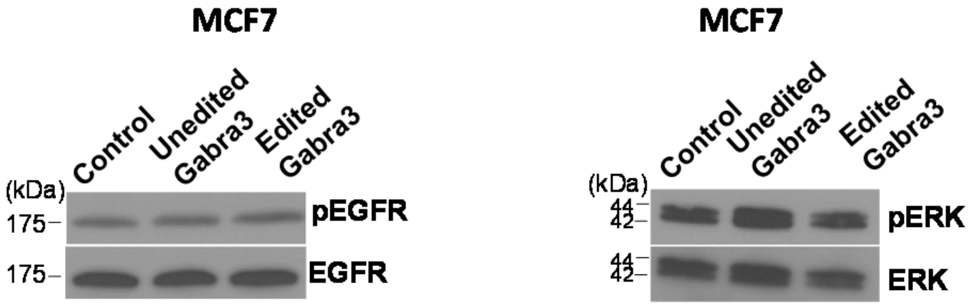

Supplementary Figure 8. Gabra3 does not affect EGFR or ERK activation Phosphorylated and total EGFR, phosphorylated and total ERK in human breast cancer MCF7 cells expressing RNA-edited Gabra3, unedited Gabra3 or a control vector, were determined by immunoblotting.

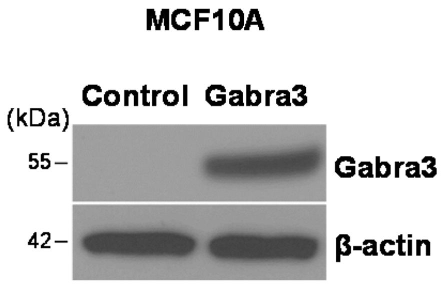

Supplementary Figure 9. MCF10A does not express endogenous Gabra3 Gabra3 expression in MCF10A cells expressing Gabra3 cDNA or a vector control were determined by immunoblotting.

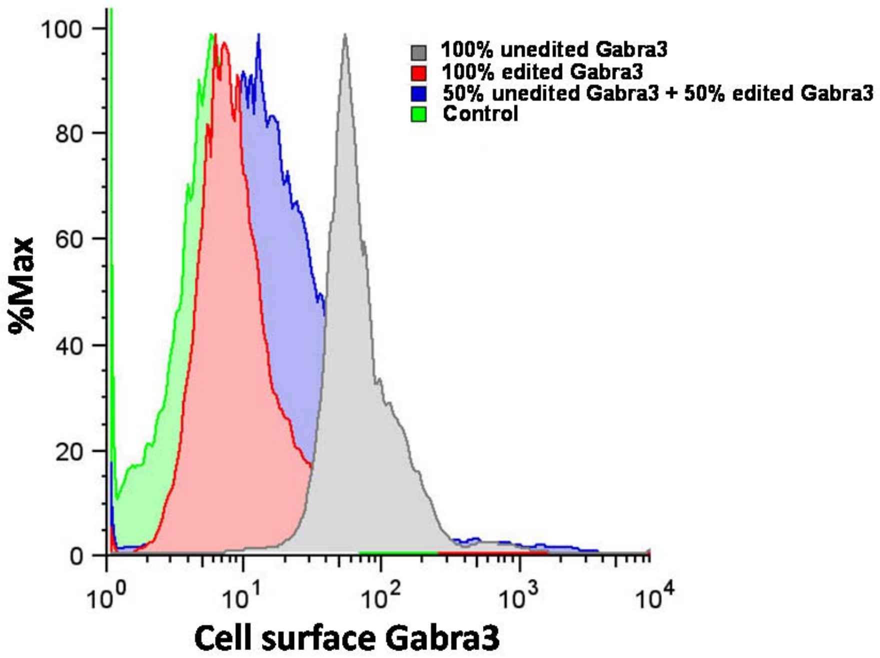

Supplementary Figure 10. Expression of unedited Gabra3 increase Gabra3 expression on cell surface, expression of edited Gabra3 reverses the phenotype Representative flow cytometry histogram overlay of Gabra3 surface expression in MCF10A cells. MCF10A cells expressing a control vector (green), or unedited Gabra3 (grey), or RNA-edited Gabra3 (red) or both unedited and edited Gabra3 at 1:1 ratio (blue), were subjected to FACS analysis using a Gabra3 antibody or control IgG.

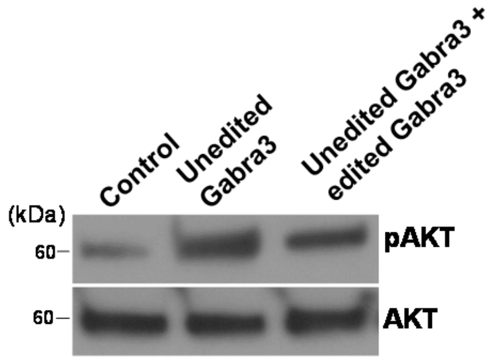

Supplementary Figure 11. The expression of unedited Gabra3 activates AKT without affecting total AKT, expression of edited Gabra3 reverses the phenotype Phosphorylated and total AKT in MCF10A cells expressing unedited Gabra3, or both unedited and edited Gabra3 at 1:1 ratio, or a control vector, were determined by immunoblotting.

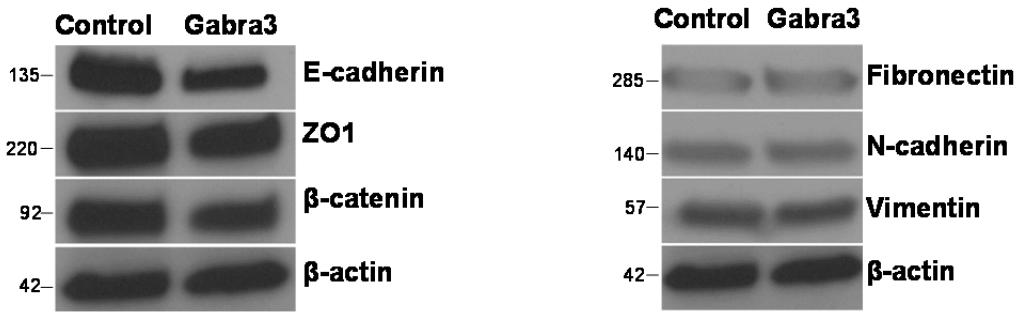

Supplementary Figure 12. Expression of unedited Gabra3 does not affect the expression of epithelial and mesenchymal markers The expression of epithelial markers E-cadherin, ZO1, β-catenin and mesenchymal markers fibronectin, N-cadherin, vimentin in MCF10A cells expressing unedited Gabra3 or a control vector were determined by immunoblotting.

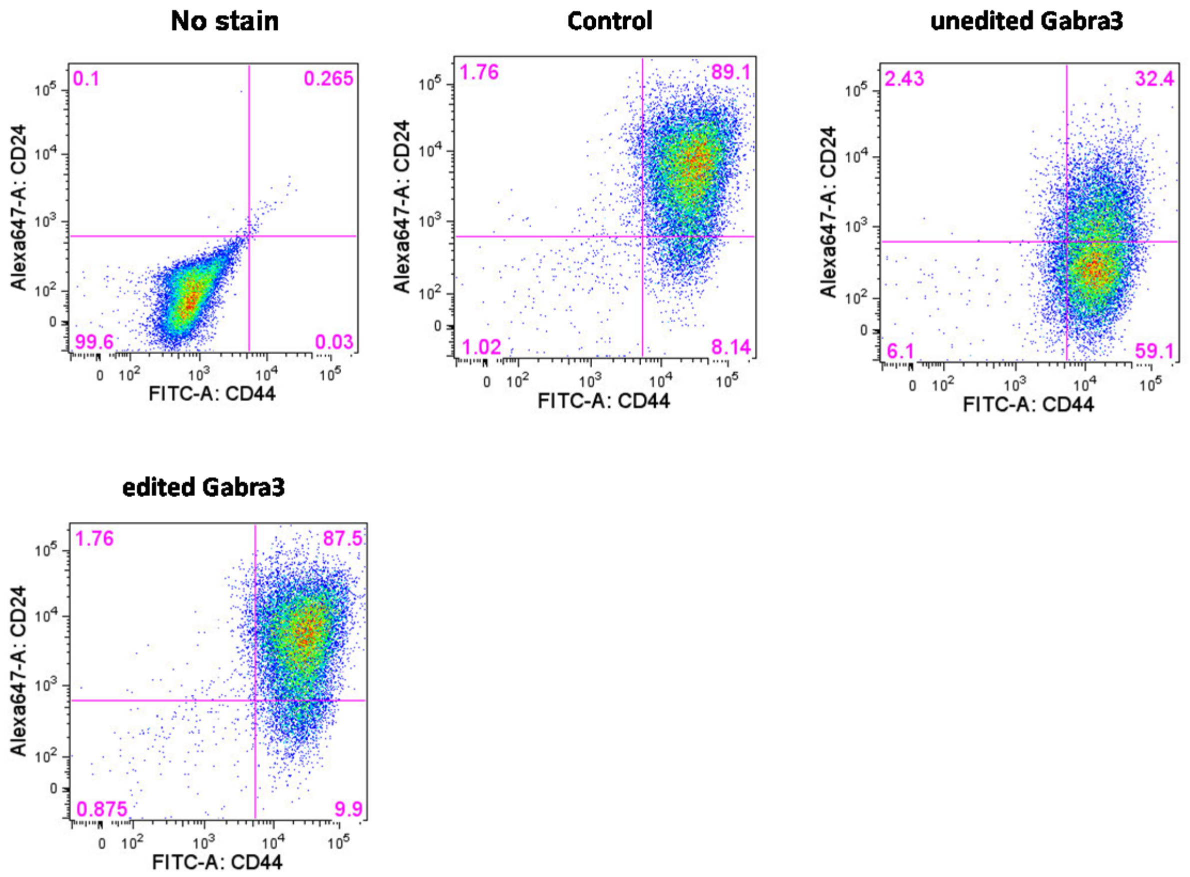

Supplementary Figure 13. Unedited Gabra3 significantly increases CD44 high/CD24 low stem cell subpopulation, whereas edited Gabra3 does not affect the percentage of stem cell population
Representative flow cytometry histogram overlay of stem cell population in MCF10A cells. MCF10A cells expressing a control vector, or unedited Gabra3, or RNA-edited Gabra3, were subjected to FACS analysis using a CD44 antibody and a CD24 antibody. Unstained and single-antibody stained cells were used as controls for setting the quadrants. Percentage of cell population with CD44 low/CD24 low, CD44 low/CD24 high, CD44 high/CD24 low, CD44 high/CD24 high was indicated. The events in the lower right quadrant represent the CD44 high/CD24 low stem cell subpopulation.

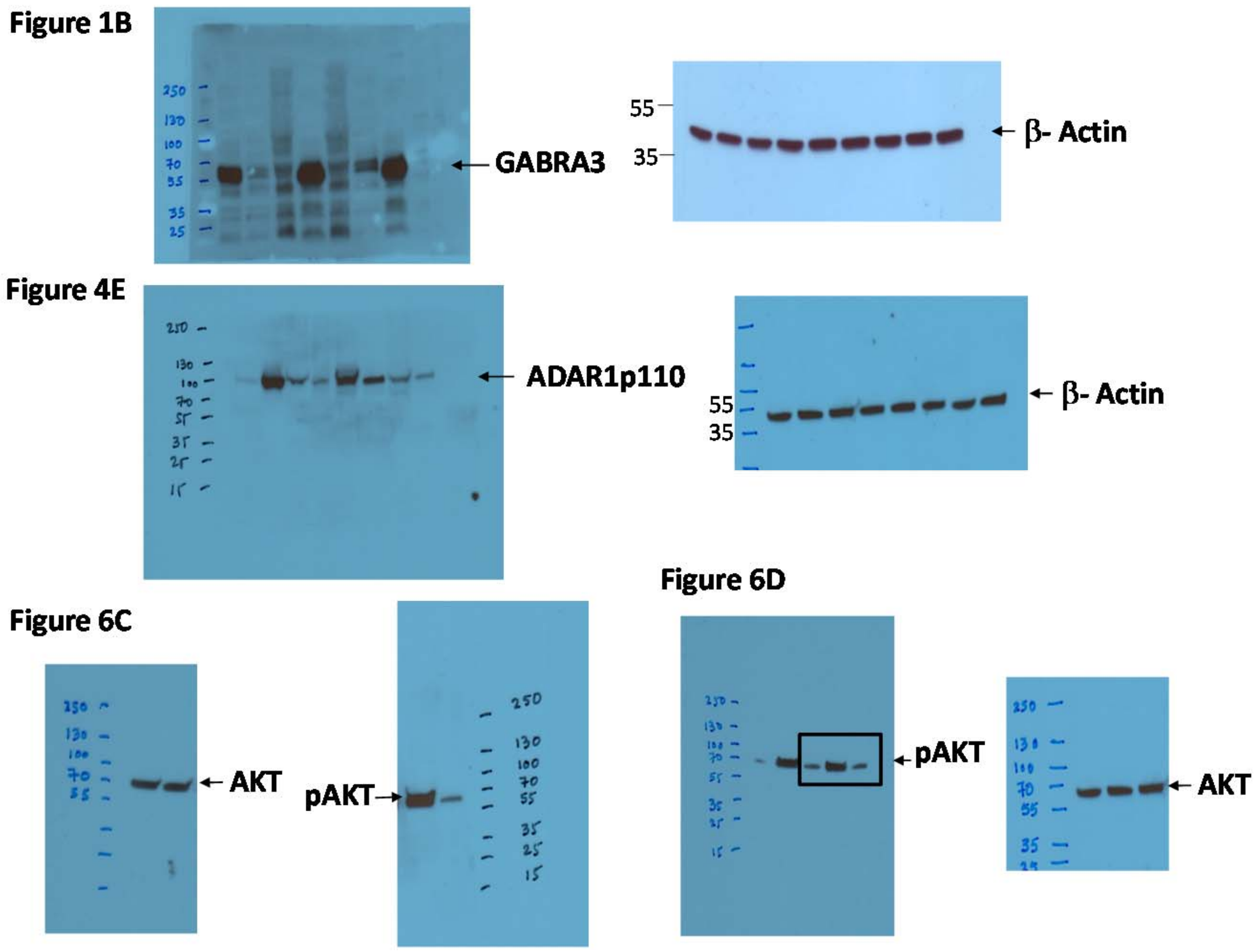

Supplementary Figure 14. Uncropped scans of Figure 1B, 4E, 6C, 6D immunoblots.

Gene	Fold increase
**PCSK1**	**88.52**
**LOR**	**69.07**
**TEX19**	**36.96**
**SBK2**	**36.12**
**GABRA3**	**35.85**
**NDST4**	**33.92**
**SOHLH1**	**32.78**
**KCNH6**	**30.72**
**DPYSL5**	**29.91**
**CEACAM5**	**24.24**
**AMTN**	**20.18**
**ATP1A3**	**18.97**
**TH**	**13.83**
**ELAVL3**	**12.94**
**ABP1**	**12.65**
**SCN1A**	**11.08**
**ONECUT2**	**9.86**
**RDH16**	**9.1**
**POU4F1**	**8.87**
**ATP2B3**	**8.83**
**TERT**	**8.32**
**NOTUM**	**8.14**
**STXBP5L**	**8.09**
**PRMT8**	**8.02**
**PEX5L**	**7.95**
**CACNA1B**	**7.14**
**DNAH11**	**6.97**
**ALG1L**	**6.29**
**TCL6**	**6.23**
**HEPHL1**	**6.21**
**KIAA0319**	**6.2**
**TBX20**	**6.16**
**B4GALNT2**	**6**
**FAM9C**	**5.82**
**FLG2**	**5.74**
**SRRM4**	**5.37**
**CPLX3**	**5.33**
**KRT79**	**5.18**
**HS3ST5**	**5.04**
**CCDC83**	**5.03**
**SFTPB**	**-26.75**

Supplementary Table 1. List of genes which expression is significantly associated with survival in breast cancer samples.

Variable	Cox regression models				
	Univariate		Paired with Gabra3		All
	pv	beta	variable.pv	Gabra3.pv	var.pv
**Gabra3**	0.001	0.005	na	na	0.017
**ER+**	0.812	-0.03	0.911	0.0010	0.412
**PR+**	0.797	0.04	0.779	0.0010	0.971
**Her2+**	0.052	0.37	0.059	0.0012	0.482
**TNBC**	0.984	-0.004	0.924	0.0010	0.662
**T**	0.0085	0.41	0.012	0.0016	na
**N**	0.00014	0.51	0.0014	0.0190	na
**M**	0.00015	1.47	0.00038	0.0022	na
**Stage**	1.12E-06	0.84	9.02E-06	0.0134	3.79E-05

Supplementary Table 2. Results of multivariate cox regression tests demonstrated that Gabra3 expression association with survival is independent from other tumor characteristics. Gabra3 expression, T/N/M stage and clinical stage were significantly associated with survival in univariate model, which still retain significance when used in pair with Gabra3 expression. Gabra3 and stage were significant independent predictors of survival in multivariate model (clinical stage was selected over T/N/M stages for the analysis). Pv = p-value from the cox regression analysis, positive/negative beta values indicate positive/negative association with survival.
